# ^89^Zr Immuno-PET Imaging of Tumor PD-1 Reveals That PMA Upregulates Lymphoma PD-1 through NF*κ*B and JNK Signaling

**DOI:** 10.1155/2022/5916692

**Published:** 2022-02-12

**Authors:** Kyung-Ho Jung, Jin Hee Lee, Mina Kim, Young Seok Cho, Kyung-Han Lee

**Affiliations:** ^1^Department of Nuclear Medicine, Samsung Medical Center, Seoul, Republic of Korea; ^2^Samsung Advanced Institute for Health Sciences & Technology, Sungkyunkwan University School of Medicine, Seoul, Republic of Korea

## Abstract

Immune therapy of T-cell lymphoma requires assessment of tumor-expressed programmed cell death protein-1 (PD-1). Herein, we developed an immuno-PET technique that quantitatively images and monitors regulation of PD-1 expression on T-cell lymphomas. *Methods*. Anti-PD-1 IgG underwent sulfhydryl moiety-specific conjugation with maleimide-deferoxamine and ^89^Zr labeling. Binding assays and Western blotting were performed in EL4 murine T-cell lymphoma cells. In vivo pharmacokinetics, biodistribution, and PET were performed in mice. *Results*. ^89^Zr-PD-1 IgG binding to EL4 cells was completely blocked by cold antibodies, confirming excellent target specificity. Following intravenous injection into mice, ^89^Zr-PD-1 IgG showed biexponential blood clearance and relatively low normal organ uptake after five days. PET/CT and biodistribution demonstrated high EL4 tumor uptake that was suppressed by cold antibodies. In EL4 cells, phorbol 12-myristate 13-acetate (PMA) increased ^89^Zr-PD-1 IgG binding (305.5 ± 30.6%) and dose-dependent augmentation of PD-1 expression (15.8 ± 3.8 − fold of controls by 200 ng/ml). FACS showed strong PD-1 expression on all EL4 cells and positive but weaker expression on 41.6 ± 2.1% of the mouse spleen lymphocytes. PMA stimulation led to 2.7 ± 0.3-fold increase in the proportion of the strongest PD-1 expressing EL4 cells but failed to influence that of PD-1+ mouse lymphocytes. In mice, PMA treatment increased ^89^Zr-PD-1 IgG uptake in EL4 lymphomas from 6.6 ± 1.6 to 13.9 ± 3.6%ID/g (*P* = 0.01), and tumor uptake closely correlated with PD-1 level (*r* = 0.771, *P* < 0.001). On immunohistochemistry of tumor sections, infiltrating CD8*α*+ T lymphocytes constituted a small fraction of tumor cells. The entire tumor section showed strong PD-1 staining that was even stronger for PMA-treated mice. Investigation of involved signaling revealed that PMA increased EL4 cell and tumor HIF-1*α* accumulation and NF*κ*B and JNK activation. *Conclusion*. ^89^Zr-PD-1 IgG offered high-contrast PET imaging of tumor PD-1 in mice. This was found to mostly represent binding to EL4 tumor cells, although infiltrating T lymphocytes may also have contributed. PD-1 expression on T-cell lymphomas was upregulated by PMA stimulation, and this was reliably monitored by ^89^Zr-PD-1 IgG PET. This technique may thus be useful for understanding the mechanisms of PD-1 regulation in lymphomas of living subjects.

## 1. Introduction

Lymphoma is a group of blood malignancies that develop from lymphocytes. Compared to B cell lymphomas, T-cell subtypes are associated with worse patient outcome [1, 2] As an example, the survival for peripheral T-cell lymphoma is five months for refractory patients and 11 months for relapsed cases [2]. Currently, there are limited effective treatment options for T-cell lymphomas, representing an unmet medical need with urgent requirement of new therapeutic strategies. Immune checkpoint inhibitor therapy is recently revolutionizing cancer management [3] with unprecedented responses in various tumors including certain types of lymphomas [4]. The immune checkpoint, programmed cell death protein-1 (PD-1), is an inhibitory receptor that downregulates the function of effector T lymphocytes [5] and plays a key role in cancer immune escape. Antibodies against PD-1 have shown effectiveness for the treatment of lymphoma tumors that have upregulated programmed death-ligand 1 (PD-L1) [6]. Given its success against various tumors including certain lymphomas, investigating the effects of immune checkpoint inhibitors in T-cell neoplasms has become a topic of research interest. Unfortunately, however, this has been met with only modest efficacy to date [7].

A major issue in applying antibody-based immunotherapy to T-cell neoplasms is the fact that the malignant cells and effector T-cells share potential therapeutic targets. T-cells respond to oncogenic stimuli by upregulating PD-1 expression, which acts as a tumor suppressor that inhibits oncogenic pathways. There is concern, therefore, that PD-1 blockade could facilitate lymphoma progression by accelerating the proliferation of T-cell clones that have activated oncogenic signaling [8]. This presents a unique problem for applying anti-PD-1 antibodies to T-cell lymphomas with strong PD-1 expression. This is exemplified by clinical trials of an anti-PD-1 antibody called nivolumab that resulted in rapid progression of adult T-cell leukemia-lymphomas [9] and T-cell lymphomas [10]. Furthermore, the development of secondary T-cell lymphoma was reported in a patient with an epithelial origin tumor after PD-1 inhibitor therapy [11].

Employing immune checkpoint inhibitor therapy for T-cell neoplasms is thus challenging because both immune effector cells and tumor cells can express PD-1. This underscores the importance of studies on PD-1 expression in the lymphoma microenvironment to support the rationale of immunotherapies and the need to monitor tumor-expressed PD-1 protein to optimize treatment efficacy. Biopsy-based immunohistochemical staining is limited by substantial inter- and intratumor heterogeneity in gene expression and the inability to assess the whole tumor [12]. Positron emission tomography (PET) can overcome these limitations, and immune-PET has been evaluated for noninvasive imaging of PD-1-expressing cells in vivo [13, 14]. However, PD-1 immune-PET studies to date have been limited to visualizing normal immune cells rather than malignant lymphoma cells.

Another important issue is that the mechanisms that regulate PD-1 expression in T-cell lymphomas and their similarity to those of effector T-cells remain elusive. T-cell activation is generally initiated by binding between specific antigens to T-cell receptor, leading to immune effector responses. Activation can also occur independently of receptor coupling or IL-2 signaling by direct stimulation with phorbol 12-myristate 13-acetate (PMA), alone or in combination with calcium ionophores [15–17]. However, it remains unknown whether PMA modulates PD-1 expression in T-cell lymphomas. The ability to monitor changes in tumor PD-1 status in a quantitative and longitudinal manner before and after exposure to major T-cell regulators should provide information to help resolve this question.

In this study, we thus developed an anti-PD-1 antibody site-specifically labeled with ^89^Zr for PET imaging. Unlike previous studies that attempted to visualize normal immune cells, we used our imaging technique to image tumor-expressed PD-1 and explored its capacity to noninvasively monitor changes of expression stimulated by PMA treatment in lymphomas of living mice. We further investigated the potential signaling pathways involved in the effect of PMA on tumor PD-1 status.

## 2. Materials and Methods

### 2.1. Cell Culture and Reagents

EL4 mouse T-cell lymphoma cells from the Korean Cell Line Bank were maintained in 5% CO_2_ at 37°C in a humidified atmosphere in RPMI 1640 media (Lonza, Basel, Switzerland) supplemented with 10% fetal bovine serum (FBS; Serena, Germany), 2 mM L-glutamine, and 100 U/ml penicillin-streptomycin. The cell line was authenticated by the Institutional Research Service and tested negative for mycoplasma. Cells were subcultured twice a week and used when 80% confluence was reached.

29F.1A12 rat monoclonal IgG, which reacts with mouse PD-1 (CD279), was from BioXcell (#BE0273; West Lebanon, NH), and deferoxamine-maleimide (DFO-Mal) was from Macrocyclics (Dallas, TX). Phorbol 12-myristate 13-acetate (PMA) and Tris(2-carboxyethyl)phosphine hydrochloride (TCEP) were from Sigma-Aldrich (St. Louis, MO). Among the primary antibodies used for Western blotting, rabbit IgG against HIF-1*α* was from Abnova (#PAB12138); those against total- (t-) and phosphorylated- (p-) nuclear factor-*κ*B (NF*κ*B) were from Cell Signaling (#8242S and #3033S); those against t-AKT and p-AKT were from Cell Signaling (#9272S and #9271S); those against t-I*κ*B*α* and p-I*κ*B*α* were from Cell Signaling (#4812S and #2859S); mouse IgG against *β*-actin was from Santa Cruz Biotechnology (#sc-47778); and that against HDAC1 was from Cell Signaling (#5356S). HRP-conjugated secondary anti-mouse and anti-rabbit IgG were from Cell Signaling, and HRP-conjugated secondary goat anti-rat IgG was from Santa Cruz Biotechnology.

### 2.2. Deferoxamine Conjugation and Site-Specific ^89^Zr Labeling

Anti-PD-1 IgG was site-specifically conjugated with DFO-Mal on sulfhydryl residues. Briefly, 2 mg of anti-PD-1 IgG underwent reduction of cysteine bonds with 100 mM TCEP (3.3 mM final concentration) for 20 min at room temperature (RT). Sulfhydryl residues of the antibody diluted in 0.1 M sodium phosphate containing 150 mM NaCl and 1 mM EDTA were conjugated with 56.4 *μ*l of 2 mM N-(3,11,14,22,25,33-hexaoxo-4,10,15,21,26,32-hexaaza-10,21,32-trihydroxytetratriacontane) maleimide (DFO-Mal) at RT for 60 min. The molar ratio of DFO-Mal to antibody was 60 : 1. ^89^Zr-oxalate (50 *μ*l; Korea Atomic Energy Research Institute) was neutralized with 25 *μ*l of 2 M Na_2_CO_3_ and mixed with DFO-Mal-conjugated anti-PD-1 IgG in 75 *μ*l of 0.5 M HEPES buffer (pH 7.5). Following 60 min of incubation at RT, the mixture was eluted through a PD-10 column with 0.25 M sodium acetate containing 0.5% gentisic acid. Fractions of 0.5 ml were collected and counted for radioactivity, and the peak activity fraction was used.

### 2.3. Polyacrylamide Gel Electrophoresis and Autoradiography

For autoradiography, unboiled ^89^Zr-PD-1 IgG was separated by electrophoresis on an 8% native PAGE gel in sample buffer that did not contain SDS or dithiothreitol. The gel was dried by DryEase® Mini Cellophane (ThermoFisher Scientific, Waltham, MA), and radioactive bands were detected by exposure of an X-ray film.

### 2.4. Radiochemical Purity and In Vitro Stability Assessment

Radiochemical purity and in vitro stability were assessed by radio-instant thin-layer chromatography (radio-iTLC) using ^89^Zr-PD-1 IgG immediately prepared or after incubation in phosphate-buffered saline (PBS) or FBS at 37°C for up to 6 days. Radio-iTLC was performed on an iTLC-SG glass microfiber chromatography paper impregnated with silica gel using 50 mM ethylene diamine tetraacetic acid (EDTA, pH 5.5) as the eluent. Under this condition, intact radiolabeled antibodies remain at baseline whereas free ^89^Zr^4+^ ions and ^89^Zr-EDTA migrate at the solvent front.

### 2.5. Cell Binding Assays

Binding experiments were performed on EL4 lymphoma cells (*n* = 3 per group) by adding 74 kBq of ^89^Zr-PD-1 IgG to the culture medium and incubating for 60 min at 37°C. Cells were then washed twice with cold PBS, lysed with 0.5 ml of 0.1 N NaOH, and measured for radioactivity on a high energy *γ*-counter. Binding specificity was evaluated by adding excess (500 nM) cold anti-PD-1 IgG. The effect of PMA stimulation on ^89^Zr-PD-1 IgG cell binding was assessed by adding varying PMA doses into the culture medium and incubation for 24 h at 37°C.

### 2.6. Preparation of Single-Cell Suspension and Lymphocyte Isolation from Mouse Spleen

Single-cell suspensions of mouse spleen were prepared following a Stem Cell Technologies protocol [18]. Briefly, spleens extracted from normal 6-week-old male C57BL/6 mice sacrificed by cervical dislocation were minced in 3 ml PBS by pressing with a syringe bar. The minced tissue solution was passed through a 70 *μ*m mesh strainer (Corning, NY) using 2× volume of PBS containing 2% FBS. Cell debris was removed by 10 min centrifugation at 1000 rpm. Red blood cells in the pellet were removed by 5 min treatment at 37°C with RBC lysis buffer (10 mM Tris–HCl (pH 7.3) containing 140 mM NH_4_Cl and 1 mM EDTA), followed by rapid neutralization with PBS containing 2% FBS and centrifugation for 10 min at 1000 rpm. The resultant single-cell suspension was finally washed twice with PBS containing 2% FBS and used for flow cytometry. Approximately 1 × 10^9^ mouse spleen cells were resuspended with 700 *μ*l FACS buffer (PBS containing 5% FBS, 1% BSA, and 0.1% sodium azide), and the lymphocyte population was isolated by FACS Aria cell sorting (BD Biosciences).

### 2.7. Flow Cytometry for PD-1 Expression on EL4 Cells and Lymphocytes

EL4 cells and mouse spleen lymphocytes in 6-well plates were incubated with 100 ng/ml of PMA for 24 h at 37°C. Cells were washed twice with FACS buffer and incubated with FITC-tagged anti-PD-1 monoclonal antibody (Invitrogen, MA, 1 : 100) for 30 min at 4°C. The cells were washed twice, resuspended with 700 *μ*l FACS buffer, and analyzed by FACS Aria cell sorting. Cell surface-expressed PD-1 was detected with a 488 nm laser as excitation channel and 530 nm wavelength fluorescence as emission detector channel.

### 2.8. Western Blotting for PD-1 and Candidate Signaling Pathways

Total cellular protein was obtained in cultured cells by applying lysis buffer containing proteinase and phosphatase inhibitor and in tumor tissues by homogenization. Briefly, cells were washed with cold PBS and solubilized at −20°C for 20 min with 200 *μ*l PRO-PREP protein extraction solution (iNtRON Biotechnology, Inc., Korea) supplemented with proteinase and phosphatase inhibitors. After centrifugation at 14,000 rpm and 4°C for 10 min, the supernatant was stored at −70°C until use.

Nuclear protein of cultured cells was prepared for NF*κ*B protein. Briefly, cells washed with cold PBS were suspended in ice-cold extraction reagent-I from Thermo Scientific (Waltham, MA). After 10 min incubation on ice, ice-cold extraction reagent-II was added. The mixture was vortexed for 5 sec and incubated on ice for another 1 min. The pellet was obtained by 5 min centrifugation at 16,000 × g, washed with cold PBS, and suspended in ice-cold nuclear extraction reagent. The mixture was vortexed for 15 sec every 10 min four times, then centrifugated at 16,000 × g for 10 min. The supernatant was finally transferred to a prechilled tube and used as the nuclear fraction extract.

Total cellular protein (20 *μ*g) and nuclear protein (10 *μ*g) were separated by 10% SDS PAGE and transferred to polyvinylidene fluoride (PVDF) membranes. The membranes were incubated at 4°C overnight with primary antibodies including rat IgG against PD-1 (1 : 1000), rabbit IgG against HIF-1*α* (1 : 2000), rabbit IgG against p-AKT (1 : 1000), rabbit IgG against p-NF*κ*B (1 : 1000), and rabbit IgG against p-I*κ*B*α* (1 : 1000). After washing with TBST buffer, membranes were incubated with HRP-conjugated secondary anti-rat IgG (1 : 2000) or anti-rabbit IgG (1 : 2000) at RT for 1 h. Immunoreactive protein was detected with enhanced chemiluminescence substrate, and band intensities were quantified. After visualizing the target protein, membranes were stripped and reincubated with mouse IgG against *β*-actin (1 : 5000), rabbit IgG against t-AKT (1 : 2000), rabbit IgG against t-NF*κ*B (1 : 2000), rabbit IgG against t-I*κ*B*α* (1 : 2000), or mouse IgG against HDAC1 (1 : 2000) as loading controls.

### 2.9. In Vivo Pharmacokinetics

All animal experiments were conducted in accordance with the National Institute of Health Guide for the Care and Use of Laboratory Animals and approved by the Institute Ethics Committee. For pharmacokinetic analysis, normal 8-week-old (20 g) male wild-type Balb/c mice were intravenously injected with 3 MBq of ^89^Zr-anti-PD-1 (*n* = 5). Serial blood from the tail vein (5 *μ*l) was measured for radioactivity as percent injected dose (%ID) per milliliter. Time activity curves were fitted by nonlinear regression with GraphPad Prism V8.4.2 using two-phase exponential decay equations. Early and late clearance rate constants (K1 and K2) and half-lives (T1/2*α* and T1/2*β*) were calculated as parameters.

### 2.10. Murine Tumor Models and PMA Treatment

T-cell lymphoma tumor models were prepared in 8-week-old (20 g) male wild-type C57Bl6 mice by a subcutaneous injection of 1 × 10^5^ EL4 cells into the right shoulder. Studies were performed when the tumor diameter reached 0.5 cm at approximately seven days after cell inoculation.

For PET imaging and biodistribution studies, tumor-bearing mice were randomly allocated into the control group, blocking group, and PMA treatment group. The blocking group received intravenous administration of excess (800 *μ*g) cold anti-PD-1 IgG 1 h preinjection. The PMA group was stimulated with 200 *μ*g/kg of PMA intraperitoneally injected, three times per week for two weeks, while the control and blocking groups were injected with the same volume of vehicle (DMSO in saline).

With respect to signaling pathway identification, separate groups of EL4 tumor-bearing C57BL/6 mice were intraperitoneally injected with 200 *μ*g/kg of PMA (*n* = 5) or vehicle (*n* = 5) every day for three consecutive days and sacrificed by cervical dislocation at 24 h after the final treatment. Tumors were extracted and underwent Western blot experiments.

### 2.11. In Vivo PET Imaging and Biodistribution Studies in Tumor Models

Each mouse was intravenously injected with 4.8 MBq ^89^Zr-PD-1 IgG via the tail vein. After 6 days, the animals were isoflurane anesthetized and underwent PET/CT imaging on a Siemens Inveon scanner. After PET/CT imaging, mice were sacrificed by cervical dislocation, and major organs and tumors were extracted, weighed, and measured for radioactivity on a high-energy *γ*-counter.

### 2.12. Immunohistochemistry for CD8*α*+ Lymphocytes and PD-1-Expressing Cells in Tumor

Microsection slides of paraffin tumor tissue underwent overnight incubation at 4°C with primary anti-mouse CD8*α* (CST, #98941S; 1 : 100) or PD-1 antibodies (Abcam, #ab214421; 1 : 500). The slides were then incubated with an anti-rabbit secondary antibody using an EnVision™ Detection System kit (peroxidase-conjugated polymer backbone, DAKO). Finally, sections were counterstained with hematoxylin and mounted with coverslips.

### 2.13. Statistical Analysis

All data are presented as mean ± SD. Significant differences between groups were analyzed by two-tailed unpaired Student's *t*-tests for two groups and by ANOVA with Tukey's post hoc test for three groups. Correlations were assessed by simple linear regression analysis. *P* values less than 0.05 were considered statistically significant.

## 3. Results

### 3.1. DFO Conjugation and Site-Specific PD-1 Antibody ^89^Zr Labeling

**Figure 1:**
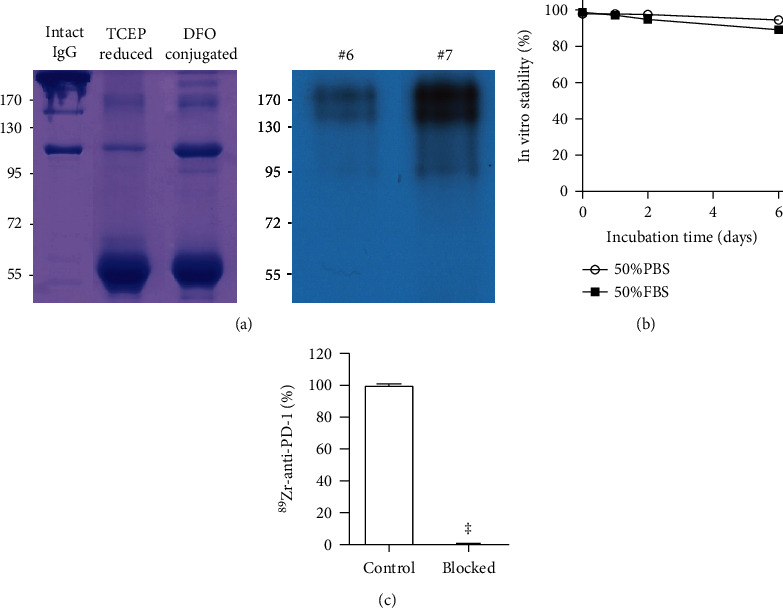
^89^Zr-PD-1 IgG autoradiography, stability, and binding specificity. (a) Autoradiography of PD-10 column-eluted fractions of ^89^Zr-PD-1 IgG on native PAGE. (b) In vitro radiochemical stability of ^89^Zr-PD-1 IgG in PBS and FBS. (c) Complete blocking of ^89^Zr-PD-1 IgG binding to EL4 cells by excess cold anti-PD-1 IgG demonstrates excellent target specificity. Bars represent the mean ± SD of triplicate samples per group. ^‡^*P* < 0.001.

29F.1A12 PD-1 IgG was site-specifically conjugated with ^89^Zr on sulfhydryl residues. Autoradiography of the first peak elute fraction after PAGE analysis displayed a clear radioactive band at the expected 170 kD region (Figure 1(a)). ^89^Zr radiolabeling was reproducible with an efficiency of >70%. The radiochemical purity was >98%, and the specific activity was 0.96 mCi/mg. Analysis by radio-iTLC showed high radiochemical stability that exceeded 95% and 89% after 6 days of incubation at 37°C in PBS and FBS, respectively (Figure 1(b)).

### 3.2. EL4 Lymphoma Cell Binding and In Vivo Pharmacokinetic Properties

Binding assays demonstrated that high ^89^Zr-PD-1 IgG binding to EL4 T-cell lymphoma cells was abolished to 0.6 ± 0.2% of that of unblocked control cells in the presence of 500 nM of cold anti-PD-1 IgG (*P* < 0.001; Figure 1(c)). This demonstrates the excellent target specificity of ^89^Zr-PD-1 IgG.

**Figure 2:**
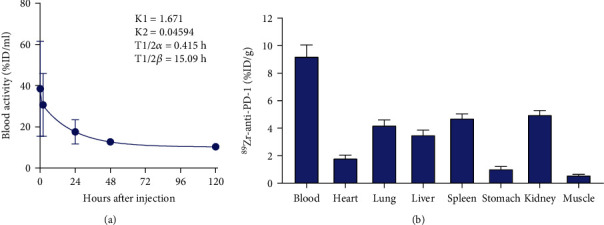
In vivo pharmacokinetics and biodistribution. (a) Time-dependent blood clearance of ^89^Zr-PD-1 IgG following intravenous injection into normal Balb/C mice. Curve fitting by two-phase decay shows biexponential blood clearance that derives pharmacokinetic parameters of early and late rate constants (K1 and K2) and half-lives (T1/2*α* and T1/2*β*). (b) Biodistribution of ^89^Zr-PD-1 IgG in normal Balb/C mice at 5 days after intravenous injection.

When intravenously injected into normal mice, ^89^Zr-PD-1 IgG was cleared from the circulation in a biexponential manner. Pharmacokinetic analysis described early K1 and late K2 rate constants of 1.671 and 0.046, respectively, that led to an early distribution half-life (T1/2*α*) of 0.42 h and late clearance half-life (T1/2*β*) of 15.1 h (Figure 2(a)).

The biodistribution of ^89^Zr-PD-1 IgG in normal mice at five days after administration showed blood activity of 9.2 ± 0.8%ID/g, followed by uptakes in the kidney at 5.0 ± 0.3%ID/g, spleen at 4.7 ± 0.3%ID/g, lung at 4.2 ± 0.4%ID/g, liver at 3.5 ± 0.4%ID/g, and low muscle uptake at 0.6 ± 0.1%ID/g (Figure 2(b)).

### 3.3. PMA Stimulates EL4 Lymphoma Cell PD-1 Expression and ^89^Zr-PD-1 IgG Binding

**Figure 3:**
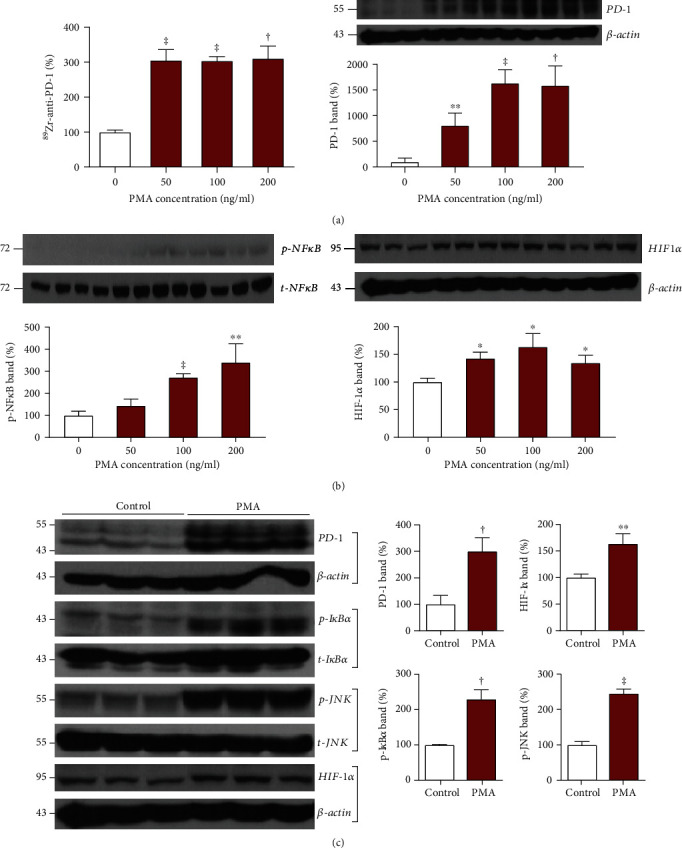
Effects of PMA stimulation on cultured EL4 cells. (a) Effects of 24 h treatment with graded doses of PMA on ^89^Zr-PD-1 IgG binding (left) and PD-1 immunoblots with *β*-actin-corrected band intensities (right). (b) Effects of graded doses of PMA on Western blots of p-NF*κ*B (left) and HIF-1*α* (right) with quantified protein band intensities that are corrected by controls. (c) Immunoblots of PD-1, HIF-1*α*, p-I*κ*B*α*, and p-JNK with quantified protein band intensities corrected by controls at baseline and following 24 h treatment with PMA. All bars represent the mean ± SD of triplicate samples per group. ^∗^*P* < 0.05, ^∗∗^*P* < 0.01, ^†^*P* < 0.005, and ^‡^*P* < 0.001.

Treatment of EL4 lymphoma cells with 50 ng/ml of PMA for 24 h stimulated a significant increase in ^89^Zr-PD-1 IgG binding to 305.5 ± 30.6% of that of untreated cells (*P* < 0.001; Figure 3(a)). Western blot analysis showed an accompaniment of dose-dependent and substantial increases in PD-1 protein that reached 15.8 ± 3.8-fold of control level by 200 ng/ml PMA (*P* < 0.005; Figure 3(a)).

When candidate signaling pathways were investigated, PMA dose-dependently increased the level of activated NF*κ*B (p-NF*κ*B) that reached 272.5 ± 16.7% (*P* < 0.001) and 340.6 ± 84.0% (*P* < 0.01) of controls by 100 and 200 ng/ml, respectively (Figure 3(b)). Treatment with PMA doses between 50 and 200 ng/ml also significantly increased HIF-1*α* accumulation to between 1.3- and 1.6-fold of the control levels (Figure 3(b)). Given these results, additional Western blots of PMA-treated EL4 cells were performed. The results confirmed that stimulation with 100 ng/ml of PMA for 24 h significantly increased PD-1 expression to 300.1 ± 51.1% (*P* < 0.005) and HIF-1*α* accumulation to 163.5 ± 23.8% (*P* < 0.01) of untreated cells (Figure 3(c)). The PMA stimulation also significantly increased p-I*κ*B*α* protein to 229.8 ± 26.5% (*P* < 0.005) and p-JNK protein to 245.1 ± 12.7% (*P* < 0.001) of untreated controls (Figure 3(c)). This indicates the potential roles of HIF-1*α*, NF*κ*B, and JNK signaling in the ability of PMA to upregulate PD-1 expression.

### 3.4. Flow Cytometry for PD-1 Expression on EL4 Cells and Lymphocytes and Effects of PMA

**Figure 4:**
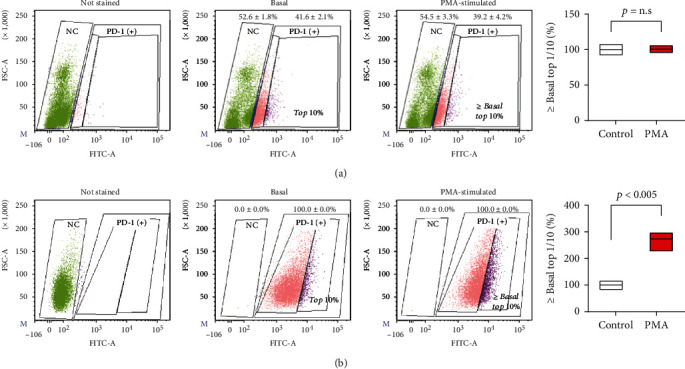
PD-1 expression on mouse lymphocytes and EL4 cells before and after PMA stimulation. Flow cytometry analysis of PD-1 expression on mouse splenic lymphocytes (a) and EL4 lymphoma cells (b). PMA stimulation was performed by treatment with 100 ng/ml for 24 h. Samples were assessed for PD-1+ rate and increase in proportion of cells with strong PD-1+ staining (upper 10% at baseline) before and after PMA stimulation. Graphs on the right hand illustrate the mean and SD of proportion of strong PD-1+ cells obtained from triplicate samples per group.

All EL4 tumor cells (100%) showed strong PD-1 expression, whereas 41.6 ± 2.1% of mouse spleen lymphocytes were PD-1+ but with lower expression levels (Figure 4). Moreover, PMA simulation led to a 2.7 ± 0.3-fold increase in proportion of the strongest (≥basal top 10% level) PD-1-expressing EL4 cells, whereas it failed to increase that of PD-1+ mouse lymphocytes (Figure 4). These results confirm that EL4 tumor cells have greater PD-1 expression than mouse lymphocytes (that include effector T-cells) and that PMA stimulation further increased PD-1 expression in EL4 cells, whereas it minimally influenced that in mouse lymphocytes.

### 3.5. Effects of PMA Treatment on ^89^Zr-PD-1 IgG PET and Biodistribution

**Figure 5:**
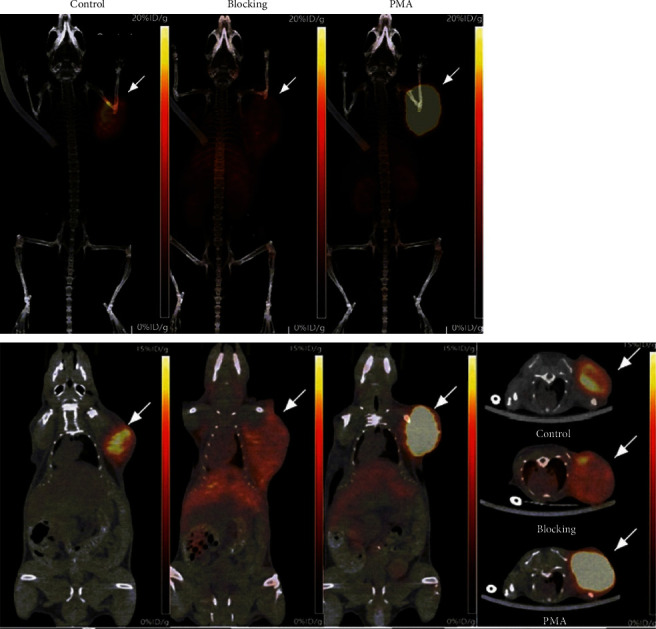
PET/CT imaging of tumor ^89^Zr-PD-1 IgG uptake and effects of PMA stimulation. Representative maximum intensity projection images (a–c) and coronal (d–f) and transaxial PET images (g) of mice at baseline, with excess antibody blocking, and after PMA treatment at 6 days after intravenous ^89^Zr-PD-1 IgG. Arrows indicate EL4 tumors.

In EL4 tumor-bearing mice, PET/CT imaging at 6 days after ^89^Zr-PD-1 IgG injection demonstrated clear tumor visualization. Tumor uptake was effectively reduced by preinjection of excess cold anti-PD-1 IgG, demonstrating specific tumor targeting of ^89^Zr-PD-1 IgG (Figure 5). Importantly, PET/CT showed that treatment with repeated intraperitoneal administration of PMA resulted in significantly increased ^89^Zr-PD-1 IgG uptake by EL4 tumors (Figure 5).

**Figure 6:**
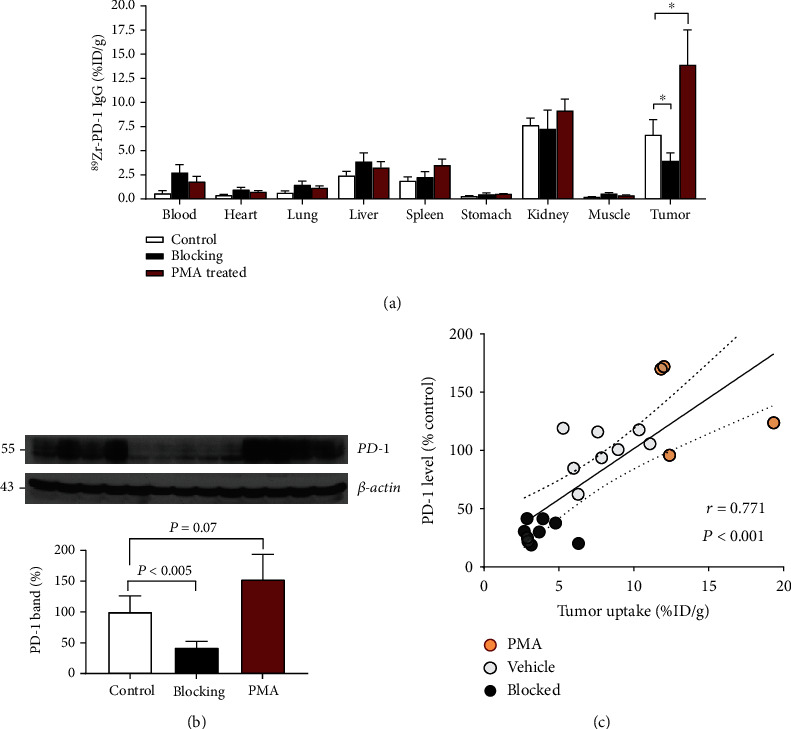
Effects of PMA on ^89^Zr-PD-1 IgG biodistribution and tumor PD-1 expression. (a) ^89^Zr-PD-1 IgG uptake in organs of EL4 tumor-bearing mice treated with vehicle (control) or PMA. The blocking group was conjected with excess cold anti-PD-1 antibody. (b) Immunoblots of tumor tissue from the above animals for PD-1 protein and band intensities corrected by *β*-actin. All bars represent the mean ± SD of quadruple mice per group. ^∗^*P* < 0.05 and ^†^*P* < 0.005. (c) Correlation between tumor PD-1 content and tumor ^89^Zr-PD-1 IgG uptake level. Data from two separate experiments include eight mice of the control group (empty circle), eight of the blocking group (filled circle), and four of the PMA group (red circle). The solid line denotes the linear regression, and dotted lines indicate the 95% confidence range.

Biodistribution studies performed immediately following PET imaging (Figure 6(a)) confirmed this finding by demonstrating significantly increased ^89^Zr-PD-1 IgG accumulation in EL4 tumors following PMA treatment (13.9 ± 3.6%ID/g) compared to that of controls (6.6 ± 1.6%ID/g; *P* = 0.01). Again, preinjection of excess cold anti-PD-1 IgG caused a 40.5% reduction in tumor uptake compared to control animals, confirming specific targeting (Figure 6(a)). Activities in the liver, spleen, and kidneys remained relatively low in vehicle- and PMA-treated animals.

### 3.6. Tumor PD-1 Is Enhanced by PMA Treatment and Correlates with ^89^Zr-PD-1 IgG Uptake

When tumors were extracted following PET imaging, Western blotting showed that PD-1 expression was significantly reduced to 42.1 ± 10.2% of the control by preinjection of excess cold anti-PD-1 (*P* < 0.005; Figure 6(b)), likely reflecting PD-1 internalization and degradation. Notably, PMA treatment was confirmed to cause a significant increase of PD-1 expression in the tumors of T-cell lymphoma model mice to 153.0 ± 40.3% compared to vehicle-injected controls (*P* = 0.07; Figure 6(b)).

When tumor PD-1 expression levels were compared to ex vivo tumor ^89^Zr-PD-1 IgG uptake levels, a close linear correlation was found (*r* = 0.771, *P* < 0.001; Figure 6(c)). This supports the reliability of noninvasive ^89^Zr-PD-1 IgG PET imaging for the quantitative assessment of PD-1 status in lymphomas of living subjects.

### 3.7. CD8*α*+ Lymphocytes and PD-1-Expressing Cells in Tumor Tissue

**Figure 7:**
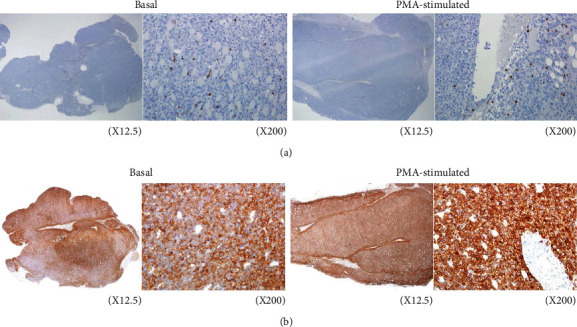
Immunohistochemistry of EL4 tumor tissue of mice with and without PMA treatment. CD8*α* staining showing tumor-infiltrating effector lymphocytes (a) and PD-1 staining of tumor cells and lymphocytes (b) in tumors of control and PMA-treated mice. Magnification, ×12.5 (left) and ×200 (right).

On microsection slides, CD8*α*+ effector T-cells were seen infiltrating the tumor, but this constituted only a very small fraction compared to malignant tumor cells (Figure 7(a)). The entire tumor section showed strong PD-1 staining that obviously included mostly EL4 tumor cells and a small fraction of PD-1+ lymphocytes. Tumor tissue of mice treated with PMA showed even stronger PD-1 staining in the entire tumor section, which, again, can be attributed mostly to EL4 tumor cells (Figure 7(b)).

### 3.8. Potential Signaling Related to the Stimulatory Effect of PMA on Tumor PD-1

**Figure 8:**
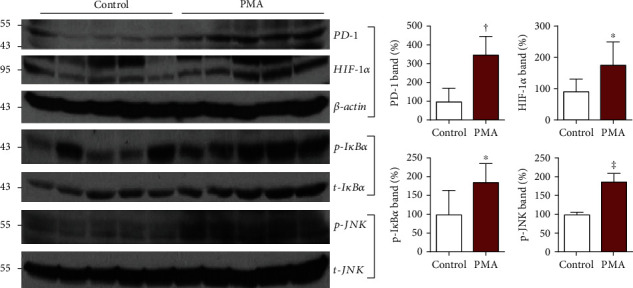
Effects of PMA on signaling pathways in EL4 tumors. Immunoblots of tumor tissue for PD-1, HIF-1*α*, p-I*κ*B*α*, and p-JNK with quantified protein band intensities corrected by controls. Animals were intraperitoneally administered with vehicle or PMA for 3 days. Bars represent the mean ± SD of quintuple mice per group. ^∗^*P* < 0.05, ^†^*P* < 0.005, and ^‡^*P* < 0.001.

Finally, further animal experiments were performed to explore potential signaling pathways involved in the ability of PMA to modulate tumor PD-1 expression. Western blot analysis of EL4 tumors demonstrated that treatment of mice with repeated PMA injection significantly increased PD-1 expression to 348.8 ± 96.5% (*P* < 0.005) and HIF-1*α* accumulation to 176.8 ± 72.3% (*P* < 0.05) of vehicle-injected mice (Figure 8). The PMA treatment also significantly increased p-I*κ*B*α* and p-JNK expression to 185.8 ± 49.1% (*P* < 0.05) and 187.3 ± 21.3% (*P* < 0.001), respectively, of control animals (Figure 8).

## 4. Discussion

PD-1 on effector T-cells function to downregulate excessive immune responses that could cause tissue damage. However, this contributes to immune escape in patients with malignant tumors [3]. Antibodies against PD-1 block this immune evasion and reactivate the immune response against various cancers. With respect to lymphoma treatment, nivolumab was approved for Hodgkin's disease and pembrolizumab was approved for Hodgkin's disease and mediastinal large B-cell lymphoma [19]. Therefore, broadening the use of this strategy for T-cell neoplasms is a desirable treatment option [20]. However, since the antibodies can also target PD-1-positive lymphoma cells, it is necessary to assess tumor PD-1 status to avoid disease progression by tumor cell activation. This prompted us to explore the usefulness of immuno-PET to identify and noninvasively assess PD-1 status in T-cell lymphomas. This has not been previously investigated because PD-1 imaging studies to date have all focused on normal immune cells rather than malignant lymphoma cells.

For our purpose, we used 29F.1A12, a rat monoclonal IgG2a that specifically binds to the extracellular domain of mouse PD-1. 10F.9G2 has been used in previous studies to investigate PD-1 expression and function by FACS analysis [21] and to block PD-1 in colon carcinoma tumors of mice [22]. There are two previous studies that used ^89^Zr-labeled anti-PD-1 antibodies. Natarajan et al. ^89^Zr labeled an anti-PD-1 human antibody called Keytruda to image lymphocytes in tumor-bearing mice [13]. More recently, van der Veen and coworkers synthesized ^89^Zr-labeled pembrolizumab and imaged its uptake in lymphoid organs [14]. However, PD-1 antibodies in these studies were used to image tumor-infiltrating lymphocytes.

In our study, we employed site-specific conjugation for ^89^Zr labeling as an established method to improve radioprobe homogeneity and preservation of immunoreactivity compared to nonspecific labeling methods. Maleimide-deferoxamine conjugation was used to direct ^89^Zr attachment to the cysteine sites of 29F.1A12. Incubation with TCEP led to cysteine site-specific reduction and DFO-antibody conjugation, likely at the two hinge region disulfide bonds [23, 24]. The conjugation technique was straightforward and required only a short 1-hour reaction time at RT. The resultant ^89^Zr-PD-1 IgG synthesized was shown to efficiently bind to EL4 lymphoma cells that express high levels of PD-1, and the binding was completely blocked by excess cold antibody, confirming excellent target specificity. Although the magnitude of increase in ^89^Zr-PD-1 IgG binding was substantial, it did not reach that of Western blot results, likely due to presence of cytosolic PD-1 protein that does not bind ^89^Zr-PD-1 IgG. Membrane proteins are synthesized in the cellular cytosol, where they remain until transported to the cell membrane. Furthermore, it is known that membrane PD-1 protein can be efficiently internalized into the cytosol [25]. PD-1 protein that is likely present in the cytosol in significant amounts could therefore cause a mismatch between changes in whole cell lysate Western blot-assessed and surface binding-assessed quantities.

^89^Zr-PD-1 IgG intravenously administered to normal mice showed favorable blood pharmacokinetics with modest activities in the liver, spleen, and kidneys after five days. Although our biodistribution data did not include bone tissue, the PET/CT images did not show any visible bone uptake for up to 6 days after ^89^Zr-PD-1 IgG injection. This contrasts with mice injected with ^89^Zr-chloride, ^89^Zr-oxalate, or ^89^Zr-phosphate in the study of Abou et al. [26], where distinct bone uptake was visible on PET images. This indicates the absence of significant amounts of free ^89^Zr in our study.

In normal mice, blood activity remained rather high at 5 days (9%ID/g). This is not unexpected, since intact antibodies have long blood half-lives that average 10 to 12 days in normal mice [27]. In contrast, EL4 tumor-bearing mice displayed significantly lower 6-day blood activity (2.5%ID/g), in a manner accompanied by high tumor uptake (13%ID/g). This indicates that high PD-1-expressing tumors efficiently extracted ^89^Zr-PD-1 IgG from the circulation, thereby acting as a sink organ that facilitated blood clearance.

The PET/CT imaging in murine models of EL4 lymphoma displayed clear tumor visualization with excellent contrast and relatively low activities in normal organs. Antibodies against PD-1 target malignant lymphoma cells but could also target effector T-cells. This was the basis for previous publications that demonstrated tumor accumulation of radiolabeled anti-PD-1 antibodies even when the tumor cells themselves did not express PD-1 [13, 14]. Immunohistochemistry of EL4 tumor tissue in our study confirmed the presence of CD8*α*+ effector T-cells infiltrating the tumor, but this constituted only a small fraction compared to malignant tumor cells. In contrast, the entire tumor section showed strong PD-1 staining, obviously representing mostly EL4 tumor cells and a small fraction of PD-1+ lymphocytes. FACS analysis also showed strong PD-1 expression on all EL4 tumor cells, whereas less than half of mouse spleen lymphocytes were PD-1+ with lower expression levels. Together, these results provide strong support that tumor uptake of ^89^Zr-anti-PD-1 was predominant by binding to PD-1-expressing EL4 tumor cells rather than to infiltrating lymphocytes.

We next investigated how PMA affects PD-1 expression and ^89^Zr-PD-1 IgG binding on cultured EL4 T lymphoma cells. The results revealed that 24 h of treatment with PMA caused significant increases in both ^89^Zr-PD-1 IgG binding and Western blot measured PD-1 expression on EL4 cells. FACS analysis also showed that PMA treatment led to substantial increases in proportion of EL4 cells with the strongest PD-1 expression, whereas the effect was minimal in mouse lymphocytes.

Treatment of EL4 tumor-bearing mice with PMA resulted in a significant enhancement in tumor uptake of ^89^Zr-PD-1 IgG without influencing uptake in other organs. Western blot analysis of the tumor tissue attributed the enhanced uptake to upregulated PD-1 expression. Immunohistochemistry confirmed that PD-1 staining that was positive throughout the entire tumor section of control mice became even stronger in tumor tissue of mice treated with PMA.

Interestingly, there were significantly lower amounts of PD-1 protein in the blocking group compared to the control group. ^64^Cu-labeled monoclonal Ab targeting the T-cell receptor was previously shown to stably label T-cells through endocytosis and internalization of the Ab-receptor complex within 24 h [28]. Furthermore, Meng et al. showed that PD-1 is internalized from the cell surface, ubiquitinated, and degraded in proteasome [25]. Together, these facts support internalization and degradation of Ab-bound surface PD-1 as a likely explanation for the lower PD-1 protein observed in the blocking group.

Importantly, there was a close correlation between magnitude of tumor ^89^Zr-PD-1 IgG uptake and tumor PD-1 protein level in vivo, confirming the reliability of immuno-PET for monitoring changes in tumor PD-1 status. The ability of PMA to stimulate PD-1 expression in lymphoma tissue has not been previously reported. Noninvasive assessment of changes in tumor PD-1 expression might allow a more rational application of immune checkpoint therapy for T-cell lymphomas.

Mechanistically, PMA is an independent activator of NF*κ*B, a transcription factor that activates T-cells and increases IL-2 production [29–31]. In its inactive state, cytosolic NF*κ*B is complexed with the inhibitory subunit I*κ*B*α*. Upon activation, I*κ*B*α* is phosphorylated and releases active NF*κ*B subunits for gene transcription [32]. A previous study showed that PMA stimulated immune T-cells via I*κ*B*α* phosphorylation and NF*κ*B activation [33]. In our results, cultured EL4 lymphoma cells exposed to PMA showed increased I*κ*B*α* and NF*κ*B phosphorylation. EL4 tumors also revealed increased I*κ*B*α* phosphorylation when animals were treated with PMA. These results demonstrate that, like effector T-cells, T-cell lymphomas are also stimulated by PMA via activation of NF*κ*B signaling.

**Figure 9:**
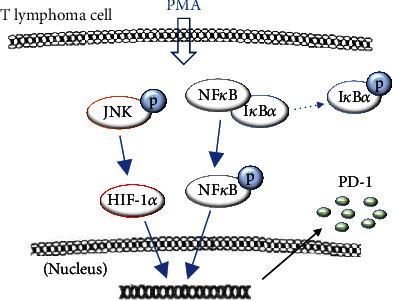
Proposed signaling mechanism for PMA-stimulated PD-1 expression on EL4 lymphomas.

Cultured EL4 lymphoma cells and EL4 tumors further showed an increase in activated JNK. Mitogen-activated protein kinases are critical for controlling the T-cell phenotype, and a role for the JNK pathway has been reported [33–35]. A previous study showed that PMA stimulated ERK and JNK activity in EL4 lymphoma cells [36]. Our findings suggest that JNK could also be involved in the ability of PMA to upregulate PD-1 in these cells.  Figure 9 illustrates a scheme of the proposed signaling pathways related to PMA-induced PD-1 upregulation.

In addition to its highly inflammatory nature, PMA is known to be a potent tumor promoter. In various tumors, increased HIF-1*α* also plays a role in tumor growth and progression. In tumor cells, PMA stimulation under normoxic conditions was shown to induce HIF-1*α* via mitogen-activated protein kinase pathways [37]. In addition, PMA has been shown to trigger crosstalk between NF*κ*B and HIF-1*α* [38]. We found that PMA led to increased HIF-1*α* accumulation in EL4 lymphoma cells and EL4 tumors, indicating that it may have a role in PD-1 expression.

## 5. Conclusion

^89^Zr-PD-1 IgG provided specific and high-contrast imaging of EL4 lymphoma tumors. Although immune T-cells infiltrating the tumor microenvironment may also have contributed, our findings indicated that tumor ^89^Zr-anti-PD-1 uptake predominantly represented binding to PD-1-expressing EL4 tumor cells. PMA stimulation was revealed to significantly upregulate tumor PD-1 expression in a manner that involved NF*κ*B, JNK, and HIF-1*α* signaling, and this was faithfully represented by increased ^89^Zr-PD-1 IgG binding in vitro and enhanced tumor uptake in vivo. Thus, ^89^Zr-PD-1 IgG PET could be useful for monitoring tumor-expressed PD-1, which in turn may help lead to more rational application of immune checkpoint therapies for T-cell lymphomas.

## Data Availability

All data generated or analyzed during this study are included in this published article.
